# A Rescued Fragment

**Published:** 1888-06

**Authors:** 


### A Rescued Fragment.

Below is a part of a manuscript article taken from a pile of miscellaneous papers tumbled out of a waste basket. It is not all here, but in the leaves before me are found some thoughts which I think worth while to send you. It reads as if a part of an article written for some periodical, but I find no printer's mark on it, nor do I recollect seeing it in print. Do with it as you think best. * *

"The man who claims to be a philosopher is said to be more modest than one who sets up as a scientist. By derivative definition a philosopher is a *lover* of wisdom—not claiming to be wise; and by the same rule, a scientist is one who *knows*.

Though, in a certain sense, science is a unit, yet it is more convenient and practical to regard it as made up of divisions and subdivisions. Moral science is not much like mathematical, and even in the division of mathematics, arithmetic, and geometry differ widely. It follows, then, that if human intellect varies in its faculties, and genius is always partial, no one man is likely to excel throughout the entire range of the sciences. Great fitness to lead in any one science, to some extent, unfits for leadership in the others. And this is a fortunate state of affairs, when properly recognized, and made practical.

Life is too short for any one to learn all that should be known. Hence, as social beings, we must one help another. Mutual help is the rule of the race in all departments of life. Many of these pertain to only our present state of existence, but knowledge is eternal in its nature, and the only way for us to make much headway in gaining it, in view of the brevity of this life, is to lay hold on the life to come, and then go on in the acquisition of wisdom and knowledge through countless ages. "Here we know in part * * * but there we shall know * * *."

It is a pity that, in our short-sighted weakness, when one of us is able to lead in a certain scientific direction, he is in danger of regarding himself as a leader and guide in all directions; and he is a pretty good fellow if he does not become impatient when contradicted. (And this thought consoles the writer, who never frets at criticism.)

To illustrate the point stated above, we might refer to the writings of ourselves and friends. We would include also our foes, if we had any, and could recall what they have written.

Our readers can easily find scores of instances in which we have written almost magisterially, in reference to subjects outside the line of our most careful studies; but we have faith in our opinions, and speak and write accordingly, claiming for them only their exact value.

Take the next illustration from a friend—a good one—yea, verily! Dr. Black. He knows more about microbes than most of us—knows very much about many things—knows still more about a few things, and yet here is a statement he makes with language fit only for positive knowledge, with assured assertion as if he were reciting the multiplication table. He calls the moon, "A dead world, where even the chemical affinities lie locked in an embrace that knows no waking, an eternal sleep;" yet of this latter he knows nothing at all. He can *guess* how it is; and so can any Yankee. We have no objection to his opinion, but when we meet in the high school above we expect him to think better of the moon. Even though it doesn't shine on dark nights, we regard it as a *fair* satellite, and rather active. It gains nearly a month on the sun each year. It turns over and over, it runs around and around, it is obedient to the force called gravitation, as modified by centrifugal force, it receives light, and gives a part, or all of it out again. As it receives light from the sun it is probable that it also receives heat—even though it be "the cold, pale moon" of the poet. With all these forces and motions, involving great changes in conditions, aside from the watchful care of its Maker, has any one a right to say, two and two are four, and the moon's chemical affinities lie locked in "an eternal sleep?" Only He who made the moon knows the secret of its slumbers.

Very many of us have learned to regard Dr. Black as *our teacher;* and just now it seems that the best use we can make of him is to demonstrate that it is not safe to blindly follow any human teacher, by a most friendly consideration of some of his teachings. In doing it we expect to be misunderstood but not by Dr. Black.

In this consideration we shall notice some thoughts in Dr. B's article in the *Dental Review*, page 653 and following. The article is headed, "THAT DEAD DOG." With very much of it we heartily agree. We do not often disagree with the doctor. It is not always safe to do it.

On page 654 we find: "The man who gives his whole energy to the study of the chemical affinities, neglecting the co-relation of life as a modifier of the presentation of element to element for the exercise of these affinities in the never-ceasing changes in nature might"—do several things. But where is the man? Never before did we hear a hint of his existence. Possibly he is a fabulous relative of "the man in the moon."

On the same page our friend asks (himself?) "What is a Salt?" and failing to define it, after a square attack, of course we must *give up* the conundrum. No one is to blame for not defining a thing not yet idealized by definite thoughts.

But look on the teachings of page 665 of the *Dental Review*. A journal alluded to the principle of elements held passive by vital force, being set free and rendered active by death, and oxygen was given in illustration. Our teacher's reply is, "Well, suppose we admit that all this is true, what of it? Do we find the meat burned to ashes? Not at all."

Now, if we had the teacher's definition of *ashes*, we might accept this "Not at all" answer. But if by "burned to ashes," he means simply thorough combustion of the material burned, it will not do. Carbonic acid escapes from decomposing dead animal bodies. Can carbon be more completely burned? Sulphuretted hydrogen is formed and escapes. Sulphur is a supporter of combustion sometimes, and has here burned the hydrogen. But don't stop here. Follow it up, and you'll find it completely burned, if not to ashes to sulphuric acid and water, and these happily united, neither caring a fig for the fact that the other was lately a portion of a dead dog.

In like manner phosphuretted hydrogen escapes from the carcass. It was not a normal constituent of the living dog, but someway the two elements find each other every time. How do they always get together, and in exactly the same relative quantities? Does the phosphorus grow on one germ-bush, and hydrogen on one adjacent? and do the storms of life dash them together, and they adhere like two snowballs? Hardly. Is it not more likely that two industrious germ-bugs seize these elements and with their giant energies, force them together? for we are told that life controls the presentation of element to element.

At any rate phosphuretted hydrogen is formed, and though lowly bred and offensive, his motto is *excelsior*, and he aspires heavenward. The phosphorus has burned the hydrogen, but follow, and you'll soon find that oxygen, as ozone, has burned both, and now purer than virgin snow, they are phosphoric acid and water, happily united, and not the least ashamed of their origin, even though born in a dead dog.

We think our friend falls short at several points on this page (655 *Review.*) In this sentence the thought is not quite clear. "Yet all seem to agree that the heat of the body is maintained by the consumption of oxygen and the formation of carbonic acid and water. When the dog is killed it grows cold. Why? Because these chemical changes which are under the domination of life have ceased."

Very often, if not generally, a dog's body is warm soon after death, and so is a man's if killed while in vigorous health. This can be accounted for only by chemical action; and the affinities are soon satisfied to such extent as to moderate the action, then the carcass becomes colder, and the microbes are probably ready by this time, and affinity accepts their aid, without an apology for not awaiting their arrival. Let a single case illustrate this point:

Some years ago a brother of the writer, a prodigy as to physical strength and vigor, was drowned on a pleasant evening in June. He was under water about an hour and a half. He had been dead over three hours when the body reached the family home. The surface over the chest and abdomen was warmer than similar surfaces of the living men present—very perceptibly warmer than the writer's, then a boy of seventeen.

The sentence next after the one quoted above is a little mystifying, as we interpret it. It seems good but mixed, and we don't believe its author holds to what we think it teaches. This is it. "Oxygen enters the lungs, and thence the blood, in which it is very loosly combined with the haemoglobin of the blood corpuscle, and from thence passes to the tissues, for which it has a stronger affinity. Hence it becomes intra-molecular, or combined with the molecule which combines the elements of the tissues. With the activities of these it is again delivered up as carbonic acid and water, waste products, still in a state of combination." Wouldn't *again* in a state of combination be clearer? How could it let go its loose hold on the haemoglobin, and take a *firm* hold on carbon and hydrogen without being *out of* a state of combination or free, at some stage of the process? Or, shorter, how can it change without changing?

But this is not the dangerous part of the sentence. Here is the poison if we understand it, and we sincerely hope we do not: "Here it (oxygen) becomes intra-molecular, or combined with the molecule which combines the elements of the tissues."

This seems to teach that oxygen taken in by respiration is appropriated to the building up of tissues, becomes a constituent of them, and is, therefore, a source of nutrition. But we don't think Dr. Black so believes; for if this doctrine were true Jeremiah's wild asses would have fattened when "they snuffed up the wind like dragons." (Jeremiah, XIV, 6.)

The oxygen found in the tissues, as one of their constituents, gets there through the assimilation of food, most of which is rich in oxygen. That taken in by respiration is as a fox entering a hen house. It goes in as a destroyer—not as a builder. By its action, and mutual action on it, we have the manifestation of chemical force, by correlation developing vital force, a nice illustration of the poet's thoughts " from death comes life."

On page 659 *Dental Reivew*, Doctor B. seems to object to oxygen getting credit for action " after the cessation of life." If through any misunderstanding he is prejudiced against oxygen, can't he credit the spunky little hydrogen that not only escapes from the wreck, but rescues her fellow passengers, nitrogen, sulphur, phosphorus, etc., not forgetting her first fond love, oxygen.

Doctor B's article in the *Review* gives ground to suspect that he doesn't like oxygen. Perhaps this is the hardest thing he says of it: " No, my friend, oxygen without the affinities of oxygen is not oxygen."

What is it then ? It has not ceased to be matter. Can it be that our revered teacher is trying to lead us back into the darkness of alchemy ? Nothing is much better recognized in science than allotropy, whether physical or chemical. To illustrate it, Webster states: " Thus ozone is an active state of oxygen, and is distinct from ordinary oxygen, which is the element in its passive state."

Phosphorus active (or ordinary) is highly combustible. It can be so changed that it burns with difficulty, yet it is still phosphorus.

A very simple experiment will show an element deprived of its affinities—rendered passive—without losing its identity. It has been often referred to, but it is so convenient, and so readily observed, that we use it again: Put a bar or rod of iron into nitric acid, somewhat concentrated; the acid is rapidly decomposed, and the iron is as rapidly oxidized. Introduce a similar bar of iron into acid of the same strength, having a piece of platinum in contact with it, letting the latter reach the acid in advance of the iron. No decomposition and no corrosion take place. Remove the little peace-maker, platinum, and still all remains quiescent. Now shall we follow our revered teacher and say, " No, my friend, (iron) without the affinities of (iron) is not (iron)?"

Well, may be it is gold, and we have found " the philosopher's stone." Bring on your iron mountains Durango and Missouri, and we'll touch them into gold.

But hold--here comes a blacksmith who insists on holding "a crowner's 'quest," and says he is the "crowner" elect, and doesn't believe in the proposed change. So he swears in a deputy, who heats and welds the bar, makes nails and rivets of it, and swears it is still iron. But, says a bacteriological philosopher, Mr. Coroner, iron without the affinities of iron is not iron; and the reply comes, you go to h—igh-school; iron is *matter*, and affinity is *force*. We'll send back the iron mountains.

Doctor B. gives good counsel when he advises the study of respiration. We have taken his advice in advance, and have devoted ten fold more time to that function than to the chemical affinities, and the former can not be understood without a tolerably clear knowledge of the latter. A man who has not drawn a breath without its being a source of pain, for a large portion of his life, has likely thought of respiration now and then.

It is not good teaching to claim that only life controls the "presentation of element to element." We hope we have misunderstood this expression; for life is only one force, and we hardly think our friend believes that other forces do not modify chemical action.

Did you ever hear of a conversation like this?

MR. QUERY.--Friend Knotsure, is your neighbor, Credule, a believer?

KNOTSURE.--Oh, yes, a firm believer.

QUERY.--Believes in the One Eternal God, and the Divine Savior?

KNOTSURE.—I think not.

QUERY.—He believes the bible, I suppose?

KNOTSURE.—Oh, no.

QUERY.—But you say he is a firm believer?

KNOTSURE.—Yes, certainly, very firm.

QUERY.—In what, then, does he believe?

KNOTSURE.—I think he believes in *an eternal*——DEAD DOG.

But Dr. Black dosn't believe in anything of the kind. He believes the biggest sheep-thief dog ever killed, can be cremated, and that a few strokes of lightning might worst his carcass considerably, even if microbe life were a myth.

We have tried before to impress on our readers the partial unreliability of microscopic observations. The microscope is a wonderful instrument, and gives wonderful illustrations, and if constructed according to the laws of nature, it always tells the truth. But is this truth always truly interpreted ? is a question forced into consideration. Some of our readers will recollect earnest disputations between Professors Atkinson and McQuillan as to what was seen through the microscope—not what each had seen elsewhere and at other times, but as to what was to be seen by their alternate lookings at the same slide. Does that show certainty of determination? Both had good eyesight, and both were familiar with the use of the instrument, and used the same one then.

Take another illustration: In an investigation of protoplasm, or something, the Prince of microscopists, Heitzmann, describes something he saw; and other experts testified that what he described was a deception caused by want of proper adjustment.

(This general statement is from memory. Can't get out to find references, but in the main it is true.)

Again, a little over a year ago, the lamented Walter A. Dunn, of world-wide fame as a microscopist, prepared hæmal crystals from a fresh drop of his own blood, and from a damaged blood clot taken from the cranium of a murdered woman. These respective crystals were put on separate slides, and when submitted to the microscope, Doctor Dunn, the experimenter, regarded the experiment as satisfactory and conclusive. To our own eyes, not unfamiliar with microscopic research, these crystals were as much alike as white beans or grains of wheat, regardless of their source. The contents of the two slides appeared to be identical. Yet two men, familiar with the microscope, at least one of them being a teacher of histology, on oath, declared that while one might be the crystals, as claimed, the others, on the different slide, had no resemblance to these crystals, no likeness to those on the other slide and gave no evidence of being other than accidental matter obtained from *debris.*

Another: Lately the trinity, Heitzman, Boedecker, and Abbott, whom we all trust and swear by, agreed that they saw something (no matter what) in or through the microscope; but George S. Allan, once our pupil, but now our teacher, and with him the microscopist of Harvard University, could not see it in that light, nor any other, even though using the same slides. When the stars fall out "in their courses," on which side shall we tallowdips rally?

In this affair of three to two, it matters not for present purposes, which is right. All that we are trying to do now is to convince our readers that microscopic revelations, as yet, can not, in many cases, be so accurately and reliably interpreted, as to warrant the positive assertions of some bacteriologists and many microscopists. And that microscopic research in reference to microbes has, as yet, all the uncertainties of similar investigation in other directions, is virtually admitted by Dr. Black when he says, on page 661 *Dental Review*: "These microbes are the smallest of microscopic objects and were, of course, unseen until very recent times." And yet they are described in all their delineations, with as much assurance and with more positive assertion than any would think of using in the description of horses and cows.

And now, dear readers, don't forget that Dr. Black is our teacher—yours and ours. He is enthroned on the tripod and will sit there, "while school keeps," unless age, infirmity, or something still more unfortunate causes him to let go, and fall off. Listen, as you have opportunity to all he says. Read, if you can what he writes. He can tell you about all that is *known* of microbes, and of much that is guessed at. And even guessing is good when it hits. But let it *hit* before risking your scientific salvation on it.

In conclusion don't blindly follow your teachers; "for if the blind lead the blind, both shall fall into the ditch." And if it will not do to "go it blind" after Dr. B. you must keep open the eyes of your understanding in all other followings. Do your own thinking so as to verify, and then believe. Recognize, too, that though the microscope is a great revealer of nature's secrets, it may be misinterpreted."

---

But here is another paper found in the same basket, which seems to be what some fellow has to say about the whole affair, after reading up both sides, and witnessing some of the results. He has probably consulted a Dutch almanac, and thinks it is time "dog-days end." He says:

"My neighbor, Mrs. Sykes, mistaking them for comic juvenile monthlies, got from a dealer in waste paper, copies of the *Dental Review* and the *Ohio Journal.* She was reading to find something to frighten her children asleep. All of a sudden she exclaimed 'Dear me! Watt-a-Black dog!' She expected the little codgers to cover their heads and begin to snore, but the boys jumped out, shouting Here Watch, Seek him Bull, while their little sister said, 'Come here Fido, poor fellow!' Mrs. S. regards the books as total failures.

But she gave me a chance to read the controversy, and I think both disputants ought "to own up," like the Scotch-Irish boy at the corn-husking. Before many of you were born there were husking-bees. Both corn-pile and company were divided into two equal parts, and a race resulted. On both sides there were husking, shouting, laughing, and *singing*—no matter what, only it must not be profane nor obscene, for the girls always listened, while their aunts and mothers boiled the beans. All of Captain A's side had given a song except the boy. "Barney," said he, "you must sing, or they'll crow over us." Barney was the beginning of a Columbian orator, but the Captain didn't know it. A song at the other side ended and the boy began promptly:

"Let us hear a new song which is not very long,
  But it's all about love and murther;
The good hunter's best dog has got lost in the fog;
  And I can not tell any further."

And none of the rest could, and even now I can't. But if Barney's song was prophetic, and this controversy is its interpretation, there is no trouble in tracing the ownership of the dog, for one of the disputants is a "good hunter," (of microbes and other small game), and he sent his "best dog" up to the lakeshore for *Review*, and that is how he got into the fog. If found again he should be placed in a kennel further south, and properly *Registered.* For what does a hunter amount to without a good dog?

But regardless of this fellow's notions, let me say, Mr. Editor, that the beans, hominy, and wild fruits of the corn-husking days developed good teeth.

